# A combined approach to establishing the timing and magnitude of anthropogenic nutrient alteration in a mediterranean coastal lake- watershed system

**DOI:** 10.1038/s41598-020-62627-2

**Published:** 2020-04-03

**Authors:** Magdalena Fuentealba, Claudio Latorre, Matías Frugone-Álvarez, Pablo Sarricolea, Santiago Giralt, Manuel Contreras-Lopez, Ricardo Prego, Patricia Bernárdez, Blas Valero-Garcés

**Affiliations:** 1 Departamento de Ecología, Pontificia Universidad Católica de Chile, Alameda 340, Santiago, Chile; 2 Instituto de Ecología y Biodiversidad (IEB), Las Palmeras 3425, Ñuñoa, Santiago, Chile; 3 Laboratorio Internacional de Cambio Global, LINCGlobal PUC-CSIC, Santiago, Chile; 4 Departamento de Geografía, Universidad de Chile, Marcoleta 250, Santiago, Chile; 5 Institute of Earth Sciences Jaume Almera (ICTJA-CSIC), C/Lluis Solè Sabaris s/n, Barcelona, E-08028 Spain; 6 Facultad de Ingeniería y Centro de Estudios Avanzados, Universidad de Playa Ancha, Traslaviña 450, Viña del Mar, Chile; 7 Instituto de Investigaciones Marinas (CSIC), C/Eduardo Cabello, 6, 36208 Vigo, Spain; 8 Instituto Pirenaico de Ecología (IPE-CSIC), Avenida Montañana, 1005, Zaragoza, 50059 Spain

**Keywords:** Element cycles, Hydrology, Limnology, Geochemistry

## Abstract

Human activities have profoundly altered the global nutrient cycle through Land Use and Cover Changes (LUCCs) since the industrial revolution and especially during the Great Acceleration (1950 CE). Yet, the impact of such activities on terrestrial and aquatic ecosystems above their ecological baselines are not well known, especially when considering the response of these systems to the intensity of LUCCs on nutrient cycles. Here, we used a multiproxy approach (sedimentological, geochemical and isotopic analyses, historical records, climate data, and satellite images) to evaluate the role that LUCCs have on Nitrogen (N) cycling in a coastal mediterranean watershed system of central Chile over the last two centuries. Despite long-term anthropogenic use (agriculture, cattle grazing) in the Matanzas watershed– lake system, these LUCC appear to have had little impact on nutrient and organic matter transfer since the Spanish Colonial period. In contrast, the largest changes in N dynamics occurred in the mid-1970s, driven by the replacement of native forests and grasslands by government-subsidized tree plantations of introduced Monterey pine (*Pinus radiata*) and eucalyptus (*Eucalyptus globulus*). These LUCC had major impacts on the transfer of organic matter (which increased by 9.4%) and nutrients (as revealed by an increase in total N) to Laguna Matanzas. Our study shows that the presence of anthropogenic land use/cover changes do not necessarily alter nutrient supply and N availability *per se* but rather it is the magnitude and intensity of such changes that produce major impact on these processes in these mediterranean watersheds.

## Introduction

Human activities have become the most important driver of the nutrient cycles in terrestrial and aquatic ecosystems since the industrial revolution^1–5^. Among these, N is a common nutrient that limits productivity in terrestrial and aquatic ecosystems^6,7^. With the advent of the Haber-Bosch industrial N fixation process in the early 20^th^ century, total N fluxes have surpassed previous planetary boundaries^2,8,9^ reaching unprecedented values (i.e. tipping points) in the Earth system especially during what is now termed the Great Acceleration (which began in the 1950s and intensified in the 1970s)^10,11^. In contrast, most dramatic changes in Land Use and Cover Changes (or LUCCs) have occurred in the last few centuries, mainly linked to forestry and agro-pastoral activities^12–14^. While most of South America is currently undergoing deforestation and land clearing (e.g., the Amazon Basin), Chile has been undergoing a process of reforestation favored by large government subsidies (Forestal Law Decree of 1974). In this context and given the Carbon Neutrality 2050 commitments that the country has acquired^15^, it is essential to understand the impacts such reforestation (which typically uses introduced species) has on the mediterranean lake-watershed dynamics of central Chile, especially when other possible drivers such as ongoing climate change (which impacts freshwater supplies^16–18^) could also be important.

The onset of the Anthropocene poses significant challenges to mediterranean regions, as these have strongly seasonal hydrological regimes and with large annual water deficits^19^. Mediterranean climates occur in many regions across the world (California, central Chile, Australia, South Africa, circum-Mediterranean regions), and provide a unique opportunity to investigate global change processes during the Anthropocene in similar climate settings but with variable geographic and cultural contexts. The effects of global change in mediterranean watersheds have been analyzed from different perspectives: hydrology^20–22^, vegetation dynamics^23–25^, sediment dynamics^26–28^, changes in biogeochemical cycles^4,29,30^, carbon storage^31^ and biodiversity^32^. A recent review showed an extraordinarily high variability of erosion rates in mediterranean watersheds, positive relationships with slope and annual precipitation and the paramount effect of LUCCs^33^. However, the temporal context and effect of LUCCs on nutrient supply to mediterranean lakes has not been analyzed in much detail.

Major LUCCs in central Chile occurred during the Spanish Colonial period (17^th^–18^th^ centuries)^34–38^ with the onset of industrialization and mostly during the mid to late 20^th^ century^39,40^. Recent copper pollution caused by 20^th^ century mining and industrial smelters has been documented in cores throughout the Andes (Laguna el Ocho and Laguna Ensueño^39^ and also from our surveys in the central valley (Batuco wetland), coastal range (Cordillera de Name) and along the coast (Bucalemito and Colejuda)^41^.

Paleolimnological studies have shown how these systems respond to climate, LUCC and anthropogenic impacts during the last millennia^42–46^. Furthermore, changes in sediment and nutrient cycles have also been identified in associated terrestrial ecosystems dating as far back as the Spanish Conquest and related to fire clearance and wood extraction practices of the native forests^34,36^. Nevertheless, pollen and limnological evidence argue for a more recent timing of the largest anthropogenic impacts in central Chile. For example, paleorecords show that during the mid-20^th^ century, increased soil erosion followed replacement of native forest by *Pinus radiata* and *Eucalyptus globulus* plantations at Laguna Matanzas, Aculeo and Vichuquén lakes^43–45,47,48^.

Lakes are a central component of the global carbon cycle. They act as a carbon sink through the mineralization of terrestrially derived Organic Matter (OM) and by storing substantial amounts of organic carbon (OC) in sediments^49^. Paleolimnological studies have shown a large increase in OC burial rates during the last century^50^, however, the rates and controls on OC burial by lakes remain uncertain, as do the possible effects of future global change and any coupled synergies with the N cycle. LUCCs, intensification of agriculture and associated nutrient loading together with atmospheric N-deposition are expected to enhance OC sequestration by lakes. Climate change has been mainly responsible for the increased algal productivity since the end of the 19^th^ century and during the late 20^th^ century in lakes from both the northern^51^ and southern hemispheres^52,53^, but many studies suggest a complex interaction of global warming and anthropogenic influences and it remains to be proven if climate is indeed the only factor controlling these transitions^54^. Alternative causes for recent N increases in high altitude lakes, such as catchment mediated processes cannot be ruled out^55,56^. Few lake-watershed systems have robust enough chronologies of recent changes to compare variations in C and N with regional and local processes, and even fewer of these are from the southern hemisphere^3,55^.

In this paper, we present a multiproxy lake-watershed study, including N and C stable isotope analyses, on a series of short cores from Laguna Matanzas in central Chile, focused on the last 200 years. Our major objectives were to reconstruct the dynamics among climate, human activities and changes in the N cycle over the last two centuries and to assess the impact of the Great Acceleration (since the mid-20^th^ century) on nutrient transfer in these systems. To independently establish the magnitude of LUCCs, we complemented our record with land use surveys and satellite studies.

## Study Site

**Figure 1:**
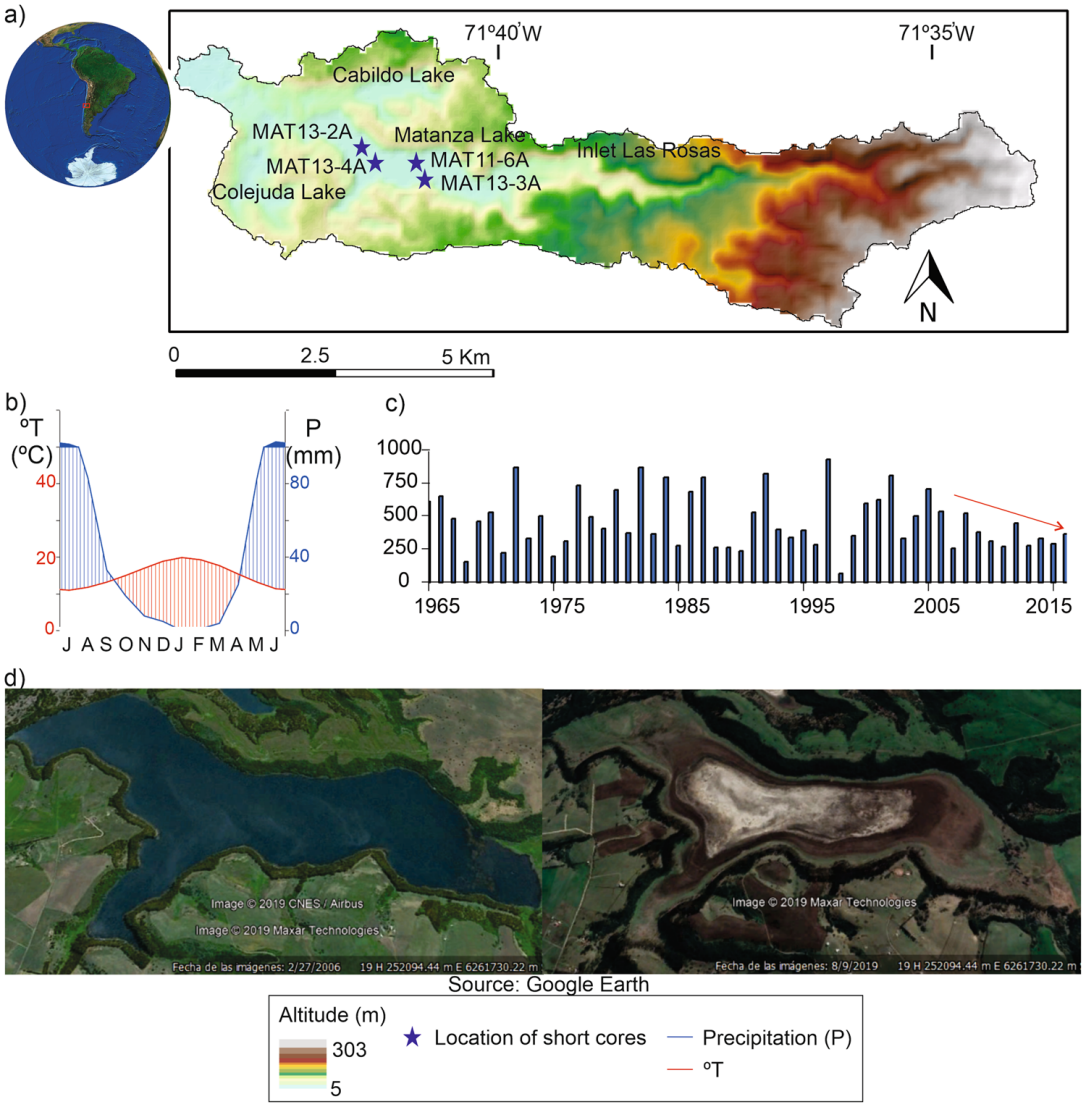
Laguna Matanzas study site. (**a**) High resolution of Digital Elevation Model (12.5 m) showing the watershed and the surface hydrologic connection with Colejuda and Cabildo lakes. (**b**) Local climograph depicting the pronounced warm dry season in austral summer and wet winters typical of mediterranean climates. (**c**) Mean annual precipitation from 1965–2015 (red arrow shows onset of the most recent “mega-drought”^89^). (**d**) A decline of lake area occurs between 2007 (left image) and 2019 (right image).

Laguna Matanzas (33°45′S, 71°40′W; 7 m a.s.l; Fig. 1) is a coastal lake located in central Chile, near a large populated area (Santiago, >6 * 10^6^ inhabitants). The lake has a surface area of 1.5 km^2^ with a max depth of 3 m and a watershed of 30 km^2^. The lake basin is emplaced over Pleistocene-Holocene aeolian and alluvial fan deposits. A recent phase of dune activity occurred from the mid to late Holocene which mostly sealed off the basin from the ocean^45^. Climate in Laguna Matanzas is characterized by cool-wet winters and hot-dry summers with annual precipitation of ~510 mm and a mean annual temperature of 12 °C. Central Chile is in the transition zone between the southern hemisphere mid-latitude westerlies belt and the South Pacific Anticyclone (SPA)^56^. In winter, precipitation is modulated by the north-west displacement of the SPA, the northward shift of the westerlies wind belt and an increased frequency of storm fronts stemming off the Southern Hemisphere Westerly Winds (SWW)^45^. Austral summers are typically dry and warm, as a strong SPA blocks the northward migration of storm tracks stemming off the SWW.

Historic land cover changes began after the Spanish conquest with a Jesuit settlement in 1627 CE near El Convento village and the development of a livestock ranch that included the Laguna Matanzas watershed. After the Jesuits were expelled from South America in 1778 CE, the farm was bought by Pedro Balmaceda who had more than 40,000 head of cattle around 1800 CE^36^. The first *Pinus radiata* and *Eucalyptus globulus* trees were introduced and planted during the second half of the 19^th^ century and were mostly used for dune stabilization^57,58^. The main plantation phase occurred 60 years ago^46^, however, as a response to the application of Chilean Forestry Laws which were promulgated in 1931 and 1974 that provided important subsidies to the industry. Major LUCCs occurred recently from 1975 to 2008 as shrublands were replaced by more intensive land uses practices such as farmland and tree plantations^59^.

Laguna Matanzas is part of the *Reserva Nacional Humedal El Yali* protected area (Ramsar N° 878). Despite this protected status, the lake and its watershed have been heavily affected by intense agricultural and farming activities during the last decades. The main inlet “Las Rosas” has been diverted for crop irrigation, causing a significant loss of water input to the lake. Consequently, the flooded area of the lake has greatly decreased in the last few decades (Fig. 1b). Exotic tree species now cover a large surface area of the watershed. Recently, other activities such as farms for intensive poultry production have been emplaced in the watershed.

## Results

### Age model

**Figure 2:**
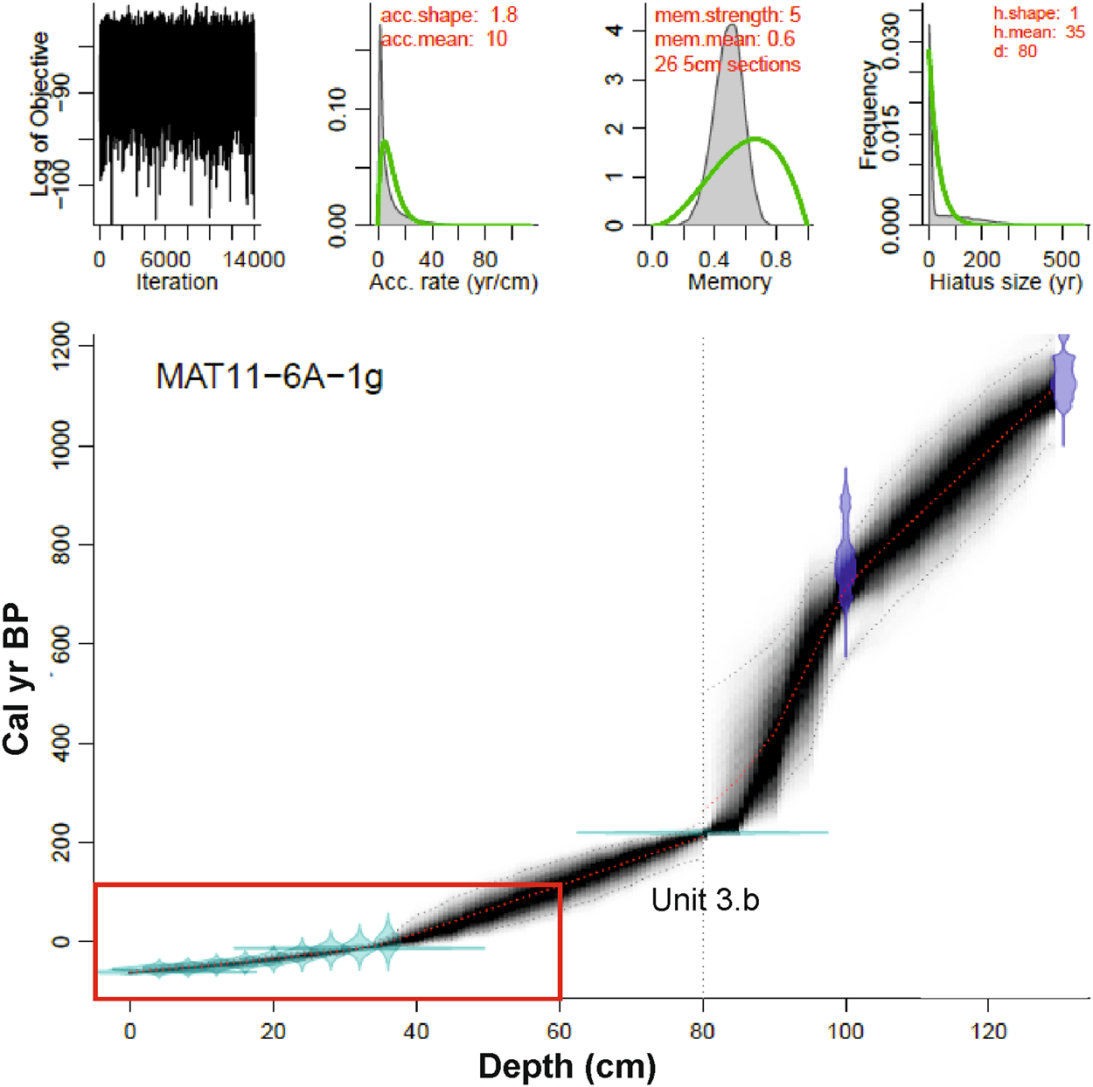
Age-depth model obtained for the Laguna de Matanzas sedimentary sequence. An instantaneous depositional event (likely a tsunami deposit) at 80 cm depth was removed for the age model (unit 3b). The section of core used for our analysis is highlighted in red.

The age model for the Matanzas sequence was developed using Bacon software to establish the deposition rates and age uncertainty^60^. It is based on ^210^Pb and ^137^Cs dates and two ^14^C dates (Table 1). According to this age model, the lake sequence spans the last 1000 years (Fig. 2). A breccia layer (unit 3.b) was deposited during the early 18^th^ century, which agrees with historic documents indicating that a tsunami impacted Laguna Matanzas and its watershed in 1730 CE^36^. Here, we focus on the last 200 years were the most important changes occurred in terms of LUCC (after the sedimentary hiatus caused by the tsunami). The Spanish colonial period (17^th^ -18^th^ century) brought new forms of territorial management along with an intensification of watershed use which remained relatively unchanged until the 1900s.

**Table 1:** Laguna Matanzas radiocarbon dates.

Lab code	Sample ID	Depth (cm)	Material	Fraction of modern C		Radiocarbon age	
				Pmc	Error	BP	Error
D-AMS 021579	MAT11-6A	104–105	Bulk Sediment	88.43	0.41	988	37
D-AMS 001132	MAT11-6A	134.5–135.5	Bulk Sediment	84.82	0.24	1268	21
POZ-57285	MAT13-12	DIC Water column		104.54	0.35	Modern

### The sedimentary sequence

**Figure 3:**
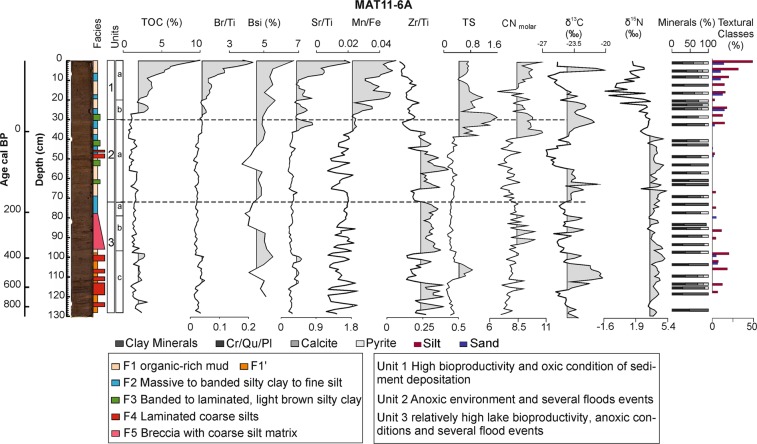
Sedimentary facies and units, mineralogy, grain size, elemental, geochemical and C and N stable isotope values of Laguna Matanzas core MAT11-6A. Values highlighted in gray indicate that these are above average. Unit 1 displayed an increase in organic matter accumulation (TOC%) associated with high bioproproductivity (Br/Ti, BioSi) under oxic lake environmental conditions (Mn/Fe).

Laguna Matanzas sediments consist of massive to banded mud with some silt intercalations. They are composed of silicate minerals (plagioclase, quartz and clay minerals) with relatively high TOC content (Fig. 3). Pyrite is a common mineral indicating dominant anoxic conditions in the lake sediments whereas aragonite occurs only in the uppermost section. Mineralogical analyses, visual descriptions, texture and geochemical composition were used to characterize five main facies (Fig. 3). F1 (organic-rich mud) represents baseline sedimentation in a shallow, well-mixed, brackish, highly productive lake and F1′ (dark orange) is a less organic facies than F1 (more details see table in the supplementary material). F2 (massive to banded silty mud) indicates periods of higher clastic input into the lake, but finer (mostly clay minerals), likely from suspension deposition associated with flooding events. Aragonite (up to 15%) occurs in both facies, but only in samples from the uppermost 30 cm and it is interpreted as endogenic, linked to higher Mg/Fe waters and elevated biologic productivity.

The banded to laminated, fining upward silty clay layers (F3) reflect deposition by high energy turbidity currents. The presence of aragonite suggests that littoral sediments were incorporated by these currents. Non-graded, laminated coarse silt layers (F4) do not have aragonite, indicating a dominant watershed sediment source. Both facies are interpreted as more energetic flood deposits but with different sediment sources. A breccia layer with coarse silt matrix and cm-long soft clasts (F5) is interpreted as a high-energy event, (i.e., a tsunami) capable of eroding the littoral zone and depositing coarse clastic material in the distal zone of the lake. Similar coarse breccia layers have been found at several coastal sites in Chile and interpreted as tsunami-related deposits^61,62^.

### Sedimentary units

Three main units and six subunits have been defined (Fig. 3) based on sedimentary facies and sediment composition. We use Zr/Ti as an indicator of the mineral fraction transported from the watershed^63^ as higher Zr/Ti (F3 and F4) is commonly associated with coarser sediments^64^. A high correlation among Br, Br/Ti, and TOC (r = 0.46–0.87, p value = 0) supports the use of Br/Ti as an indicator of lake productivity^45,63^. The Mn/Fe ratio is indicative of lake bottom oxygenation^65^, as under reducing conditions Mn mobilizes more than Fe, leading to a decreased Mn/Fe ratio^64^ Sr/Ti indicates periods of increased aragonite formation, as Sr is preferentially included in the aragonite mineral structure^66^ (see Supplementary Material).

The basal unit (3.c; 129 to 99 cm) is relatively organic-rich (TOC mean = 2.6%; BioSi mean = 5%) and composed by F1 (without aragonite) with some coarser F4 flood layers (Zr/Ti mean = 0.25). Unit 3.b (98 to 80 cm) is interpreted as a tsunami or storm surge deposit (breccia F5 grading into massive to banded silt F2). Unit 3.a (79 to 73 cm) is characterized by relatively low productivity (TOC = 2%, Br/Ti = 0.02, BioSi = 4%) under anoxic conditions (Mn/Fe = 0.01). Unit 2.a (72 to 31 cm) has relatively less organic content and more intercalated clastic facies F3 and F4. The top of this unit (43-30 cm) has elevated TS values. The Subunit 1.b (30–20 cm) shows increasing TOC, BioSi and Br/Ti values (TOC mean = 2.9%, BioSi mean = 5.4%, Br/Ti mean = 0.04) and the upper subunit 1.a (19-0 cm) has the highest TOC (mean = 6.4%) and BioSi (mean = 5.6%) high Br/Ti mean = 0.10 and the presence of aragonite. More frequent anoxic conditions (Mn/Fe lower than 0.01) during units 3 and 2 shifted towards more oxic episodes (Mn/Fe = 0.03) in unit 1 (Fig. 3).

### Isotopic signatures

**Figure 4:**
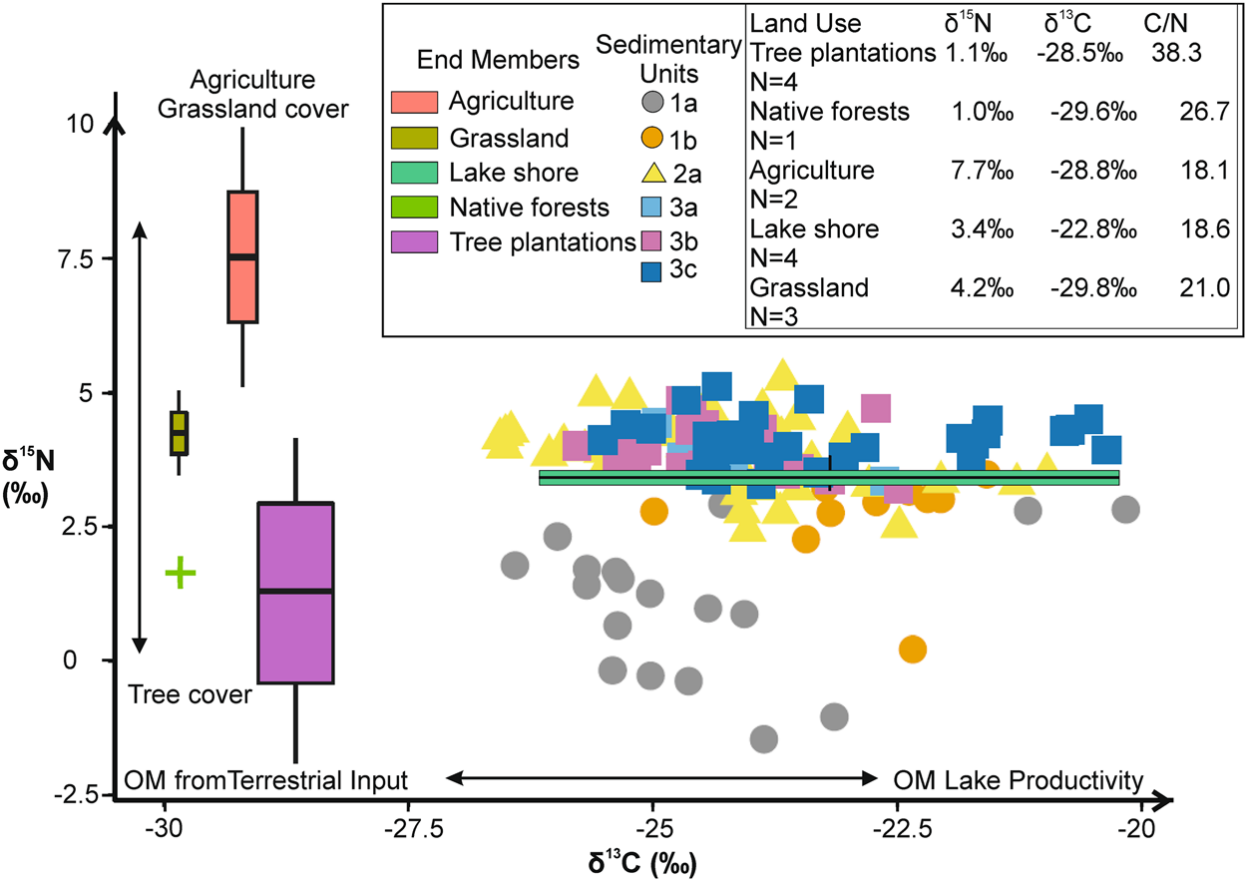
C-N stable isotope plot showing a comparison of lake sediments grouped by sedimentary units (core MAT11-6A) showing the present-day soil end members (lake shore and land use/cover) from Laguna Matanzas used to interpret the stable isotope record. The δ^13^C from modern terrestrial samples oscillate around more negative values than aquatic samples while more positive δ^15^N values are associated with agriculture and grassland cover.

Figure 4 shows the isotopic signature from soil samples of the major land uses/cover present in the Laguna Matanzas used as an end member in comparison with the lacustrine sedimentary units. δ^15^N from cropland samples exhibit the highest values, whereas grassland and soil samples from lake shore areas have intermediate values (Fig. 4). Tree plantations and native forests have similarly low δ^15^N values (+1.1 ± 2.4‰,). All samples (except those from the lake shore) exhibit low δ^13^C values (from −28.5‰ to −29,8‰). C/N_molar_ from agriculture land, lakeshore area, and non-vegetation areas samples display the lowest values (about 18). C/N_molar_ from tree plantations and native forest have the highest values (38.3 and 26.7 respectively).

The δ^15^N values from sediment samples (MAT11-6A) range from −1.5 and +5.3‰ (mean = +3.5 ± 0.5‰); δ^13^C values range from −26.6‰ to −20.2‰ (mean = −24.0, ±1.4). In Unit 3.c, δ^15^N and δ^13^C show relatively high values (mean = +4.1 ± 0.4‰ and −23.3 ± 1.5‰ respectively). δ^15^N and δ^13^C from Unit 3.b and 3.a fluctuate at slightly lower values than in 3.c (mean δ^15^N from 3.a = +3.8 ± 0.3‰ and 3.b mean = +3.9 ± 0.5‰; mean δ^13^C from 3.a = −24.2 ± 0.8‰ and 3.b mean = −24.4 ± 0.9‰). In Unit 2.a, δ^15^N values are relatively high (mean = +3.8 ± 0.7‰) but show a slightly decreasing trend (from +5.2 to +2.4‰ at 66 cm and 36 cm respectively). δ^13^C also decreases (mean = −24.2 ± 1.3‰) reaching minimum values at 45 cm (−26.6‰) with a sharp increase towards the top of this unit with maximum values ca. −21,0‰. Unit 1 exhibits the lowest δ^15^N values (1.a mean = +1.1 ± 1.3‰; 1b mean = +2.8 ± 0.9‰) with negative values in the uppermost sediments (−0.4‰ at 14 cm). δ^13^C values show a decreasing trend over most of subunit 1.b and increase only near the very top of this Unit.

### Recent land use changes in the Laguna Matanzas watershed

**Figure 5:**
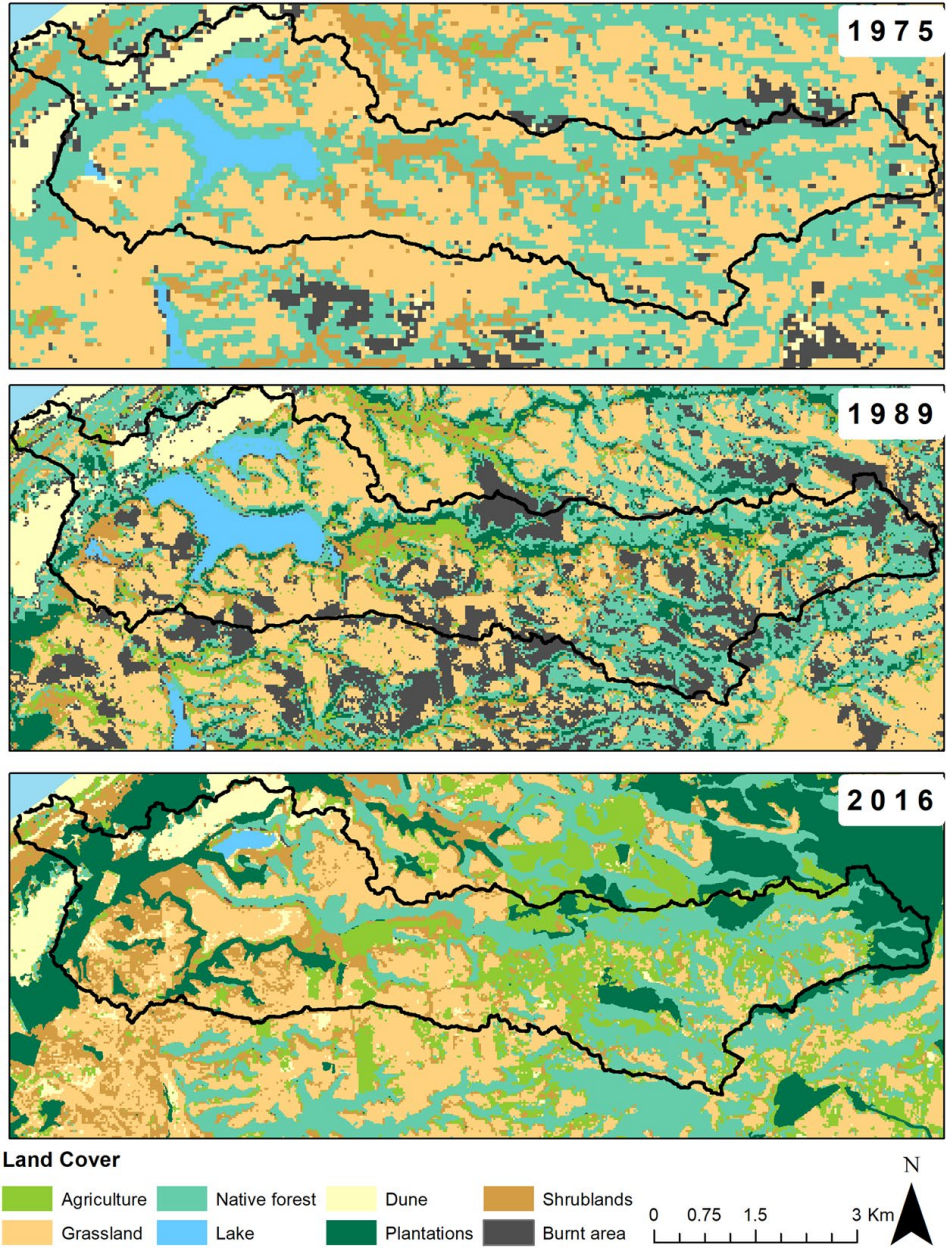
Land Uses and Cover Changes (LUCCs) derived from the analysis of the satellite imagery from 1975 to 2016 in Laguna Matanzas watershed showing changes from native forests and areas for livestock grazing (grassland) to the expansion of agriculture and forest plantation. The lake completely dried out by 2016.

Major LUCC from 1975 (Unit 1.b) to 2016 CE in the Laguna Matanzas´s watershed is summarized in Fig. 5. The watershed has a surface area of 30 km^2^, of which native forest (36%) and grassland areas (44%) represented 80% of the total surface in 1975. The area occupied by agriculture was only 0.2% and tree plantations were absent. Isolated burned areas (3.3%) were located mostly in the northern part of the watershed. By 1989, tree plantations surface area had increased to 5%, burned areas to 17%, and agricultural fields to 9% (a 45-fold increase) and native forest and grassland sectors decreased to 23% and 27%, respectively. By 2016, agricultural land and tree plantations have increased to 17% of the total area, whereas native forests decreased to 21%.

## Discussion

### N and C dynamics in laguna matanzas

Small lakes with relatively large watersheds such as Laguna Matanzas typically have relatively high contributions of allochthonous C to sediment OM^67^. Terrestrial C_3_ plants (δ^13^C from −26 to −28‰)^68,69^ are dominant in the Laguna Matanzas watershed. Likewise, our soil samples ranged across similar, although slightly more negative values (δ^13^C = −30 to −28‰, Fig. 4) to those previously proposed^69^ and are used here as terrestrial end members. Soil samples taken from the lake shore (δ^13^C = −22 ± 5‰) and POM from surface water (δ^13^C = −24‰) were more positive than the terrestrial end member and are used as lacustrine end members. Thus, more negative δ^13^C values in lake sediment samples are attributed to higher OM inputs from terrestrial vegetation and more positive δ^13^C values have increased aquatic OM^67,70^. Phytoplankton preferentially uptake ^12^C, leaving the DIC pool enriched in ^13^C especially when there are no important external sources of C (e.g., decreased C input from the watershed)^67^. During events of elevated primary productivity phytoplankton uptakes ^12^C until its depletion and are then obligated to use the heavier isotope, resulting in an increase in δ^13^C. Changes in lake productivity thus greatly affect the C isotope signal, with high productivity leading to elevated δ^13^C values^67,70,71^.

In a similar fashion, the N isotope signatures in Laguna Matanzas reflect a combination of factors, including different N sources (autochthonous/allochthonous) and lake processes such as productivity, isotope fractionation in the water column and sediment denitrification. Elevated δ^15^N values from a POM sample (+22‰) and average values from exposed lake shore sediments (δ^15^N = +3.4 ± 0.28‰) are used as aquatic end members whereas terrestrial samples have values from +1.0 ± 2.4‰ (tree species) to +7.7 ± 3.5‰ (agriculture) and represent terrestrial end members (Fig. 4).

Autochthonous OM in aquatic ecosystems typically displays low δ^15^N values when the OM comes from N-fixing species. Atmospheric fixation of N_2_ by cyanobacteria results in OM δ^15^N values close to 0‰^72^. Phytoplankton preferentially uptake ^14^N from Dissolved Inorganic Nitrogen (DIN) in the water column and derived OM typically have δ^15^N values lower than DIN values. When productivity increases, the remaining DIN becomes depleted in ^14^N, which in turn increases the δ^15^N values of phytoplankton over time, especially if the N is not replenished^70^. Thus, high POM δ^15^N values from Laguna Matanzas reflect elevated phytoplankton productivity with a ^14^N depleted DIN. In addition, N-watershed inputs also contribute to high δ^15^N values. Heavily impacted watersheds by human activities are often reflected in isotope values due to land use changes and associated modified N fluxes. For example, the input of N runoff derived from the use of inorganic fertilizers and agricultural development leads to the presence of elevated δ^15^N (between −4 to +4‰) values in the water bodies^71,73,74^. Nitrate concentrations from manure measured in Brittany (France) were reported to exhibit a positive correlation with elevated δ^15^N values^75^.

Post-depositional diagenetic processes can further affect C and N isotope signatures. Several studies have shown a decrease in δ^13^C values of OM in anoxic environments, particularly during the first years of burial related to the selective preservation of OM depleted in ^13^C^70,76–79^. Diagenetic processes can also lead to post-burial N isotope enrichment of the sediments. Indeed, ^14^N is consumed more rapidly than ^15^N by denitrifying bacteria, which intensifies under anoxic conditions^74^. Thus, the remaining OM pool will be enriched in ^15^N, in turn leading to elevated δ^15^N values^80,81^.

In summary, the relatively high δ^15^N values in sediments of Laguna Matanzas reflect N input from an agriculture/grassland watershed with positive synergetic effects from increased lake productivity, enrichment of DIN in the water column and, most likely, post-burial denitrification. The increase of algal productivity associated with increased N terrestrial input and/or recycling of lake nutrients (and lesser extent biological fixing atmospheric N) and denitrification under anoxic conditions can all increase δ^15^N values (Fig. 3). In addition, elevated lake productivity without C replenishing (e.g. by terrestrial C input) produces shifts towards positive δ^13^C values whereas C input from the watershed generates more negative δ^13^C values.

### Recent depositional evolution of the Laguna Matanzas watershed

**Figure 6:**
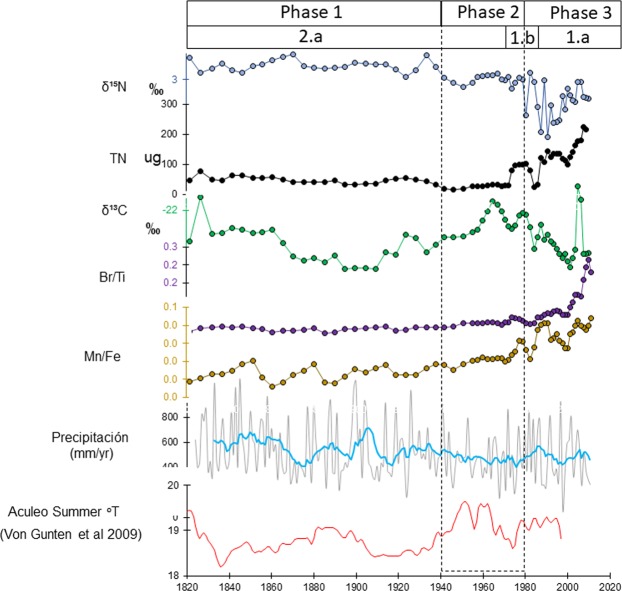
Anthropogenic and climatic forcing and Laguna Matanzas dynamic responses (productivity, sediment input, N and C stable isotopes) over the last two centuries along with mean annual precipitation and summer temperature reconstructions for central Chile^90^. Significant human impact occurs in the lake during phase 3, characterized by high productivity (Br/Ti), more negative δ^15^N values and high Total Nitrogen (TN) content under a dry-humid environment.

Sedimentological, compositional and geochemical indicators show three depositional phases in the lake evolution under the human influence in the Laguna Matanzas over the last two hundred years. Although the record is longer (close to 1000 years- see Fig. 2), we analyzed the last two centuries (Unit 2 and 1, Figs. 3 and 6) to provide a detailed historical context for the large changes detected during the 20^th^ century. Indeed, changes over the last 1000 years appear to have been of much lesser magnitude than those that occurred in the last 50 years (Figs. 3 and 4) and provide an ecological baseline against which recent impacts can be compared.

The first phase of N cycling lasted from the beginning of the 19^th^ century until c. 1940 (Unit 2.a, Figs. 3 and 6) and was characterized by moderate productivity with elevated sediment input from the watershed as indicated by our geochemical proxies (Br/Ti = 0.02, Al/Ti > 0.1). The δ^15^N values are high (~4.1‰) whereas TN exhibits moderately low values (~47.8 μg; Fig. 6). Higher lake levels and dominant anoxic bottom conditions (Mn/Fe = 0.01) are associated with increased precipitation (557 mm/yr) and lower temperatures (summer annual temperature <19 °C). Lake productivity, sediment input and elevated precipitation (Fig. 6) all suggest that N was sourced in the watershed. The N from cow manure and soil particles would have led to higher δ^15^N values in the lake^73^. In addition, anoxic lake bottom conditions would have led to even further enrichment of buried sediment N. The δ^13^C values lend further support to our interpretation of increased sediment input (and N) from the watershed. Decreased δ^13^C values reach their lowest values in the entire record (−27.0‰) at ca. 1910 CE (Figs. 3 and 6).

During most of the 19^th^ century, human activities in Laguna Matanzas were similar to those present during the Spanish Colonial period, characterized by agricultural and farming livestock development. However, the appearance of *Pinus radiata* and *Eucalyptus globulus* pollen c. 1890 CE^58^, the dune stabilization-afforestation program which began c. 1900 CE^57^ and the application of the Chilean Forestry Law Decree of 1931 (DFL n°265), contributed to an increased capacity of the surrounding vegetation to retain nutrients and sediments. State subsidized forest plantations occurred in areas devoid of vegetation and the cutting of forest on slopes greater than 45° was prohibited. These LUCCs were coeval with decreased sediment inputs (low Al/Ti) from the watershed, slightly increased lake productivity (Br/Ti from 0.01 to 0.03) and a decrease in annual precipitation (Fig. 6). N isotope values become more negative during this period, although they remained high (from +4.9 to +3.7‰) whereas the δ^13^C trend towards more positive values reflects changes in the N source from watershed to in-lake dynamics (e. g. increased endogenic productivity).

The second phase started after 1940 and is clearly marked by an abrupt change in the general trend of δ^15^N (which previously oscillated around +4.1 ± 0.4‰) to +3.2 ± 1.4‰. Overall variability in δ^15^N trends decreases together with TN (c. 43.2 μg) and low sediment input (low Al/Ti) from watershed, decreased rainfall (318 mm/yr) and a slight increase in lake productivity (increased Br/TI). These shifts in depositional dynamics thus likely mark a turning point in the lake as human activity intensified throughout the watershed and lake levels decreased.

At the onset of the Great Acceleration, Laguna Matanzas δ^15^N values shifted towards even lower values reaching c. 3‰, with an increase in δ^13^C values that do not appear to be related to increased lake productivity (i.e., Br/Ti slightly increase). The δ^13^C trend towards more positive values, peaking in the 1960s (at −21.2‰) were coeval with elevated mean summer temperature and a drop-in precipitation. A shift in OM origin from macrophytes and watershed input influences to increased lake productivity could thus explain this trend (Figs. 4, 1b).

In the 1970s, the Laguna Matanzas’ watershed was mostly covered by native forest (36%) and grassland areas were intended for livestock grazing (44%; Fig. 5). Soil OM samples from these environments show δ^15^N_Soil_ < 5‰ (Fig. 4). Farming fields occupied a very small area and tree plantations were almost nonexistent. The decreasing trend in δ^15^N values seen in our record is interrupted by several large peaks and high NT that occurred between the mid-1970s to early 1980s, when the native forest and grassland areas fell by 23% and 27% respectively, largely due to fires affecting 17% of the forests (Fig. 5). Agriculture fields increased by 4% and forest plantations increased by 9% (Unit 1.a). Concomitantly, sediment inputs from the watershed decreased (as indicated by the trend in Al/Ti), and precipitation remained relatively low (Fig. 6). These changes are likely related to the increase of vegetation cover, especially of tree plantations (that exhibit more negative δ^15^N values, Fig. 4). Although the tree cover retains much more OM, sediment and nutrient than agriculture and livestock farming, the fertilizer use for improving to yield tree plantations can be responsible for the high NT (with a low δ^15^N) in the lake sediment.

The third phase started in the 1980s (Unit 1.a) when OM accumulation rates increase and δ^13^C, δ^15^N values decrease, reaching their lowest values in the sequence c. 2000 CE. During the 21^st^ century, δ^13^C and δ^15^N values again increase along with the highest TN seen in the record (~126.6 μg). The onset of Unit 1 is marked by increased lake productivity and decreased sediment input (Al/Ti < 0.2) synchronous with intensive farming replacing forestry and extensive agriculture (Figs. 5 and 6). The increase of forest plantations was mostly in response to the implementation of the Law Decree of Forestry Development (DL 701, 1974) that heavily subsidized forest plantations. The increase in agricultural land (17% in 2016) is synchronous with increasing δ^15^N, δ^13^C, TOC and Mn/Fe trends despite the decline in rainfall and overall lower lake levels as more water is used for irrigation. The decrease in δ^15^N values until 1990 (Unit 1.a) is concomitant with decreases in native forest and grassland areas, which fell to 23% and 27% respectively, and is thus likely due to deforestation. Agriculture surface area increased to 4% and forest plantations increased to 9% of the total watershed (Fig. 5). Sediment inputs from the watershed thus decreased and are related to lower rainfall (Fig. 1.b) and an increase of vegetation cover, especially of tree plantations (which exhibit more negative δ^15^N values, see Fig. 4).

At present, agriculture and tree plantations occupy approximately 34% of the watershed, whereas native forests and grassland cover 21% and 25%, respectively. Lake productivity as indicated by Br/Ti (Fig. 6) is higher which generates OM with lower δ^15^N and higher δ^13^C values (about +2‰ and −20‰, respectively) as well as elevated TN. Even though agricultural lands and tree plantations appear to be retaining much more sediment than grasslands, the elevated TN (with relatively low δ^15^N values) thus points to an inefficient use of nitrogen fertilizers as a possible cause (Fig. 6).

## Conclusions

**Figure 7:**
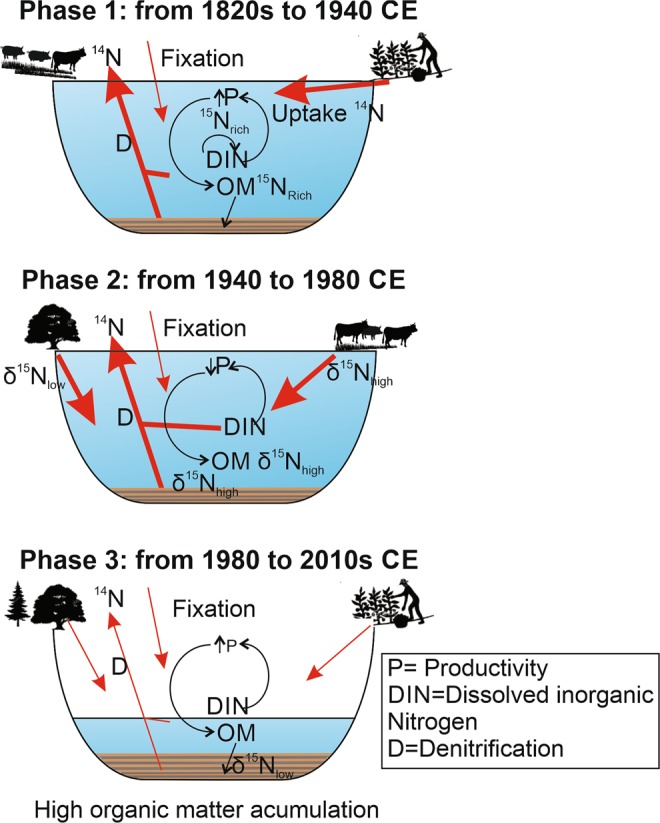
Schematic diagrams illustrating the main factors controlling the N isotope values in sediment OM of Laguna Matanzas. N input from the watershed depends on human activities and land cover type. Agriculture practices and cattle farms (grassland development) contribute more N to the lake than native forest and plantations. Periods of higher productivity tend to deplete the ^14^N in dissolved inorganic N, leading to higher δ^15^N values on OM. Post-burial denitrification processes are more effective during periods of anoxic conditions associated with higher lake levels.

Higher δ^15^N and lower δ^13^C values are associated with increased nutrient input from the watershed due to increased livestock grazing and agriculture pressures (phase 1, Fig. 7a) whereas lower δ^15^N values and high TN occurred during periods of increased forest plantations (phase 2 and 3, Figs. 6 and 7.b, c). During periods of increased lake productivity, δ^15^N values decreased well below the ecological baseline of the record as established over the last 1000 years (Fig. 3).

Furthermore, N isotope signatures in the lake sediments not only reflect changes in the watershed fluxes, but also in-lake processes such as productivity and post-depositional modifications. Indeed, post-burial denitrification could be a dominant process during periods of increased anoxic conditions, which appear to have been much more frequent before 1950.

Finally, and despite more than 200 years of human activities in the Laguna Matanzas watershed, these only appear to have been a major driver of the N and C cycle in the last 50 years. The increase in forest plantations and agriculture in conjunction with managerial decisions (that likely included fertilizer mixtures) seem to be the most important factors that are responsible for increased lake productivity, accumulation of OM and increased TN, which furthermore exhibits the lowest δ^15^N values of the entire period analyzed. We conclude that landscape anthropogenization by itself does not necessarily alter the supply of nutrients and N availability beyond the natural ecological baseline. Rather it is the magnitude and intensity of human activities over the last few decades that have produced a more pronounced impact in this mediterranean watershed.

## Methods

Short sediment cores were recovered from Laguna Matanzas using an Uwitec gravity piston corer in 2011 and 2013 (Fig. 1a). Sediment cores (MAT11-6A, 129 cm; MAT13-2A, 149 cm; MAT13-3A, 33 cm; MAT13-4A, 99 cm) were split, photographed, sub-sampled and stored at the Pyrenean Institute of Ecology (IPE-CSIC, Spain). Core MAT11-6A was obtained from the central sector of the lake and was selected for detailed multiproxy analyses (including elemental geochemistry, C and N isotope analyses, XRF, and ^14^C dating).

The isotope analyses (δ^13^C and δ^15^N) were performed at the Laboratory of Biogeochemistry and Applied Stable Isotopes (LABASI-PUC, Chile), using a Delta V Advantage IRMS coupled to a Thermo Flash 2000 Elemental Analyzer via a Conflo IV interface. Isotope results are expressed in standard delta notation (δ) in per mil (‰) relative to the standards Pee Dee Belemnite (Vienna Pee Dee Belemnite) for C and to atmospheric N_2_ for N^72^. Sediment samples for δ^13^Corg were pre-treated with 50 ml of HCl and 50 ml of deionized water and dried at 60 °C for 4 hr to remove carbonates^82^.

Total Carbon (TC), Total Organic Carbon (TOC), Total Inorganic Carbon (TIC) and Total Sulphur (TS) were measured every cm with a LECO SC 144 DR at IPE-CSIC. XRF measurements were carried out every 4 mm in MAT11-1A core using an AVAATECH X-ray Fluorescence II core scanner at the University of Barcelona (Spain). Results are expressed as element intensities in counts per second (cps). Tube voltage was operated at 30 kV and 10 kV to obtain the abundances of 15 elements (Al, Si, S, Cl, K, Ca, Ti, V, Mn, Fe, Br, Rb, Sr, Y, Zr) with an average at least of 1600 cps (less for Br = 1000).

Biogenic silica content, mineralogy and grain size were measured every 4 cm. Biogenic silica was measured following^83,84^, using an Auto Analyzer Technicon AAII for dissolved silicate analysis. Mineralogy was analyzed with a Siemens D-500 ×-ray diffractometer (Cu kα, 40 kV, 30 mA, graphite monochromator) at the ICTJA-CSIC (Spain). Grain size analyses were performed in a Beckmann Coulter LS 13 320 Particle Size Analyzer at the IPE-CSIC. The samples were classified according to textural classes as follows: clay (<2 μg), silt (20-2 µm) and sand (>2 µm) fractions.

The age-depth model for the Laguna Matanzas sedimentary sequence was constructed using ^210^Pb and ^137^Cs dating techniques (MAT13-4A, see the age model in Supplementary material) as well as two 14 C AMS dates on bulk sediment samples (MAT11-6A, Fig. 2). We dated the dissolved inorganic carbon (DIC) in the water column and no significant reservoir effect is present in the modern-day water column (104.54 + 0.35 pcmc, Table 1). ^210^Pb and ^137^Cs dates from MAT13-4A was transfer to MAT11-6A using the compositional analysis of Total Carbon (see supplementary material). An age-depth model was obtained with the Bacon R package to estimate the deposition rates and associated age uncertainties along the core (Blaauw and Christen, 2011).

To estimate LUCC of Laguna Matanzas´s watershed, we use satellite images Landsat MSS for 1975, Landsat TM for 1989 and Landsat OLI for 2016, all taken in summer or autumn (Table 2). We performed supervised classification of land uses (maximum likelihood algorithm) for each year (1975, 1989 and 2016) and the results were mapped using software ArcGIS 10.2 in 2017.

**Table 2:** Landsat imagery.

Satellite Images	Acquisition Date	Resolution
Landsat MSS	1975/03/22	60 m
Landsat TM	1989/02/17	30 m
Landsat OLI	2016/04/04	30 m

Surface water samples were filtered for obtained particulate organic matter. In addition, soil samples from the main land use/cover present in the Laguna Matanzas watershed were collected. Elemental C, N and their corresponding isotopes from POM and soil were obtained at the LABASI and used here as end members.

Daily precipitation at Santo Domingo (33°39′S 71°36′W)- the nearest weather station to Laguna Matanzas– was compiled using the redPrec R package^85,86^ (Fig. 1d). To extend our precipitation reconstruction back to 1824 we correlated this dataset with that available for Santiago. The Santiago data was compiled from data published in the Annals of Universidad of Chile^87^ for the years 1824 to 1849, Almeyda^88^ for the years 1849 to 1864 and the Quinta Normal series from 1866 to the present (Dirección Meteorológica de Chile). We generated a linear regression model between the present-day Santo Domingo station and the compiled Santiago data with a Pearson coefficient of 0.87 and p-value < 0.01.

## Supplementary information

Supplementary material.

## Data Availability

Requests for materials should be addressed to: magdalena.fuentealba@gmail.com and/or clatorre@bio.puc.cl.
