# Supplementary material for: A combined approach to establishing the timing and magnitude of anthropogenic nutrient alteration in a mediterranean coastal lake- watershed system
Source: Sci Rep. 2020 Apr 3;10:5864. doi: 10.1038/s41598-020-62627-2 (PMC7125103; doi:10.1038/s41598-020-62627-2)

Supplementary material

A combined approach to establishing the timing and magnitude of anthropogenic nutrient alteration in a mediterranean coastal lake-watershed system

Magdalena Fuentealba[abc]*, Claudio Latorre[abc]*, Matías Frugone-Álvarez[abc], Pablo Sarricolea[d], Santiago Giralt[e], Manuel Contreras-Lopez[f], Ricardo Prego [g], Patricia Bernárdez [g]; Blas Valero-Garcés[c,h]

[a] Departamento de Ecología, Pontificia Universidad Católica de Chile, Alameda 340, Santiago, Chile.

[b] Institute of Ecology and Biodiversity (IEB), Las Palmeras 3425, Nuñoa, Santiago, Chile.

[c] Laboratorio InternZacional de Cambio Global, LINCGlobal PUC-CSIC, Spain.

[d] Departamento de Geografía, Universidad de Chile, Marcoleta 250, Santiago, Chile.

[e] Institute of Earth Sciences Jaume Almera (ICTJA-CSIC), C/Lluis Solè Sabaris s/n, Barcelona, E-08028, Spain.

[f] Facultad de Ingeniería y Centro de Estudios Avanzados, Universidad de Playa Ancha, Traslaviña 450, Viña del Mar, Chile.

[g] Instituto de Investigaciones Marinas (CSIC). C/Eduardo Cabello, 6. 36208 Vigo, Spain.

[h] Instituto Pirenaico de Ecología (IPE-CSIC), Avenida Montañana, 1005, Zaragoza 50059, Spain.

*Corresponding authors
E-mail address:
clatorre@bio.puc.cl
magdalena.fuentealba@gmail.com

The age-depth model for the Laguna Matanzas sedimentary sequence was constructed using ^{210}Pb and ^{137}Cs dating techniques in core MAT13-4A as well as two 14C AMS dates on bulk sediment samples (MAT11-6A, fig. 2). We dated the dissolved inorganic carbon (DIC) in the water column and no significant reservoir effect is present in the modern-day water column (104.54 + 0.35 pcmc, Table 1). The age model for MAT11-6A was build using 14C dates and ^{210}Pb and ^{137}Cs dates from MAT13-4A correlated using the Total Carbon. An age-depth model was obtained with the Bacon R package to estimate the deposition rates and associated age uncertainties along the core (Blaauw and Christen, 2011).

Table 1. Depth/ Activity values, estimated ages and sediment accumulation values for ^{210}Pb and ^{137}Cs in core MAT13-4A

a) ^{210}Pb depth/ activity

Top of Interval	Base of Interval	Mid of Interval	Total ^{210}Pb	Error of Total Pb	Cum. Dry Mass	Unsup. ^{210}Pb	Error of Unsup Pb	Age: Base of Int.	Error of Age	Date: Base A.D.	Date: Mid A.D.	Sediment DMAR	Error of DMAR
(cm)	(cm)	(cm)	(pCi/g)	(±s.d.)	(g/cm2)	(pCi/g)	(±s.d.)	(yr)	(±s.d.)			(g/cm2 yr)	(±s.d.)
4	5	4.5	0.815	0.027	0.934	0.436	0.047	6.50	3.87	2006.5	2006.8	0.1307	0.0184
8	9	8.5	1.032	0.034	1.217	0.653	0.051	9.47	4.07	2003.5	2004.0	0.0803	0.0101
12	13	12.5	1.069	0.030	1.559	0.690	0.048	14.30	4.49	1998.7	1999.4	0.0657	0.0088
16	17	16.5	0.903	0.027	1.951	0.524	0.047	19.91	5.05	1993.1	1993.8	0.0727	0.0116
20	21	20.5	0.756	0.024	2.471	0.377	0.045	26.51	5.85	1986.5	1987.4	0.0828	0.0165
24	25	24.5	0.705	0.022	3.172	0.326	0.044	35.64	7.38	1977.4	1978.7	0.0730	0.0179
28	29	28.5	0.579	0.018	3.900	0.200	0.042	44.60	9.21	1968.4	1969.4	0.0892	0.0301
32	33	32.5	0.483	0.018	4.584	0.103	0.042	50.60	10.73	1962.4	1963.0	0.1412	0.0722
36	37	36.5	0.559	0.020	5.475	0.180	0.043	61.73	14.53	1951.3	1953.4	0.0603	0.0286
40	41	40.5	0.595	0.019	7.078	0.216	0.043						
52	53	52.5	0.326	0.010									
76	77	76.5	0.431	0.013									
92	93	92.5	0.434	0.014									

Supported Pb-210: 0.3793 ± 0.0381 pCi/g	Cum. Unsup. Pb-210: 2.2253 pCi/cm2
Number of Supported Samples: 3	Unsup. Pb-210 Flux: 0.0705 pCi/cm2 yr

b) ^{210}Pb depth/ activity

Depth (cm)	Btm Interval	Det.	Sample ht (cm)	% Inorg	^{210}Pb (Total) Not Ht Corrected	^{214}Pb (Supported) Not ht Corrected	^{137}Cs Not Ht Corrected	^{210}Pb (Total) Ht Corrected	^{214}Pb Ht Corrected	^{137}Cs Ht Corrected
**4-5**	4.5	1	2.10				0			0.0000
**8-9**	8.5	1	2.40				0			0.0000
**12-13**	12.5	1	3.00				0			0.0000
**16-17**	16.5	1	3.10				0.01			0.0082
**20-21**	20.5	1	3.00				0			0.0000
**24-25**	24.5	1	3.00				0.06			0.0485
**28-29**	28.5	1	3.00				0.14			0.1132
**30-31**	30.5	1	3.00				0.242			0.1958
**31-32**	31.5	1	3.50				0.213			0.1870
**32-33**	32.5	1	3.00				0.19			0.1537
**33-34**	33.5	1	3.50				0.161			0.1414
**34-35**	34.5	1	3.50				0.104			0.0913
**36-37**	36.5	1	3.00				0.12			0.0971
**40-41**	40.5	1	3.20				0			0.0000
**44-45**	44.5	1	2.80				0			0.0000
**48-49**	48.5	2	2.50				0			0.0000

Figure S1. ^{210}Pb and ^{137}Cs age model for MAT13-4A

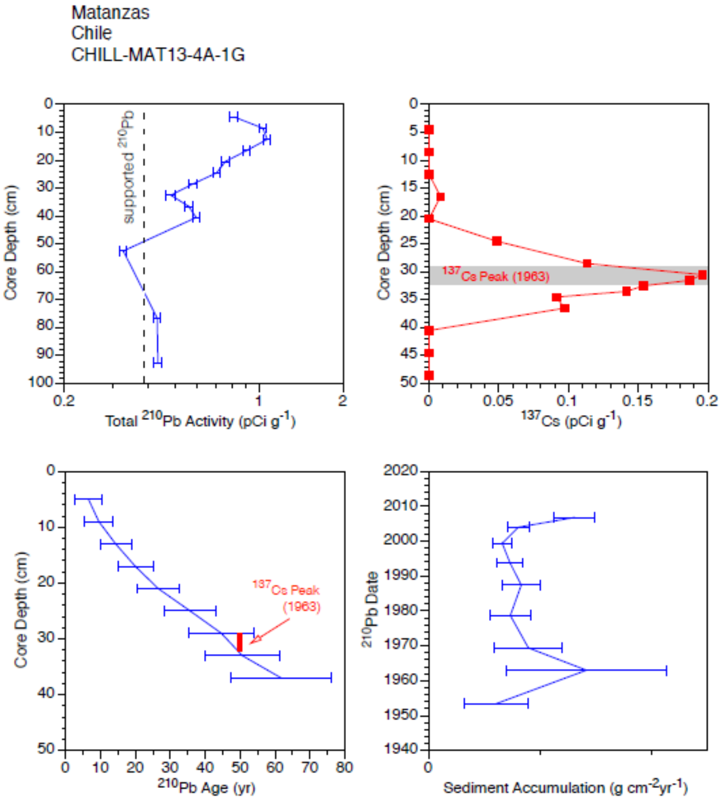

Figure S2. Correlation between the MAT13-4A and MAT11-6A cores based on Total carbon percentages

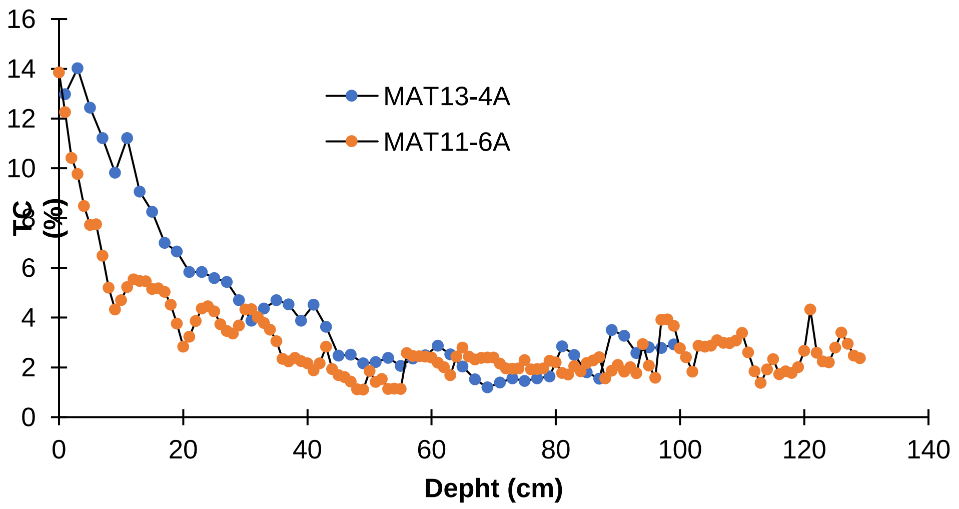

Supplement: Supplementary file 1 — Supplementary material. [file 41598_2020_62627_MOESM1_ESM.pdf]
